# Na_2_Ti_3_O_7_@RF@Ag Heterostructures as Efficient Substrates for SERS and Photocatalytic Applications

**DOI:** 10.3390/molecules29010218

**Published:** 2023-12-30

**Authors:** Yu-Cheng Chang, I-Chun Lin, Ning-Chien Chin, Sin-Ei Juang, Chia-Man Chou

**Affiliations:** 1 Department of Materials Science and Engineering, Feng Chia University, Taichung 40724, Taiwan; a0931776422@gmail.com (I.-C.L.); a111079@mail.tsmh.org.tw (N.-C.C.); juang5251@cgmh.org.tw (S.-E.J.); 2 Department of Orthopedics, Antai Tian-Sheng Memorial Hospital, Antai Medical Care Corporation, Pingtung 92842, Taiwan; 3 Department of Anesthesiology, Kaohsiung Chang Gung Memorial Hospital, Chang Gung University College of Medicine, Kaohsiung 83301, Taiwan; 4 Department of Surgery, Taichung Veterans General Hospital, Taichung 40705, Taiwan; 5 College of Medicine, National Yang Ming Chiao Tung University, Taipei 11221, Taiwan; 6 Department of Post-Baccalaureate Medicine, National Chung Hsing University, Taichung 40227, Taiwan

**Keywords:** one-dimensional heterostructures, sodium titanate nanowires, resorcinol–formaldehyde, silver nanoparticle, surface-enhanced Raman scattering, rhodamine B, photocatalysts

## Abstract

A multi-step procedure was effectively employed to synthesize innovative three-dimensional (3D) heterostructures encompassing sodium titanate (Na_2_Ti_3_O_7_) nanowire cores, an intermediate resorcinol–formaldehyde (RF) layer, and outer silver (Ag) nanoparticle sheaths, referred to as Na_2_Ti_3_O_7_@RF@Ag heterostructures. Initially, a one-step hydrothermal technique facilitated the direct growth of single-crystal Na_2_Ti_3_O_7_ nanowires onto a flexible Ti foil. Subsequently, a two-step wet chemical process facilitated the sequential deposition of an RF layer and Ag nanoparticles onto the Na_2_Ti_3_O_7_ nanowires at a low reaction temperature. Optimal concentrations of silver nitrate and L-ascorbic acid can lead to the cultivation of Na_2_Ti_3_O_7_@RF@Ag heterostructures exhibiting heightened surface-enhanced Raman scattering (SERS), which is particularly beneficial for the detection of rhodamine B (RhB) molecules. This phenomenon can be ascribed to the distinctive geometry of the Na_2_Ti_3_O_7_@RF@Ag heterostructures, which offer an increased number of hot spots and surface-active sites, thereby showcasing notable SERS enhancement, commendable reproducibility, and enduring stability over the long term. Furthermore, the Na_2_Ti_3_O_7_@RF@Ag heterostructures demonstrate remarkable follow-up as first-order chemical kinetic and recyclable photocatalysts for the photodecomposition of an RhB solution under UV light irradiation. This result can be attributed to the enhanced inhibition of electron–hole pair recombination and increased surface-active sites.

## 1. Introduction

Surface-enhanced Raman scattering (SERS) greatly amplifies the Raman signals of analytes near enhancing materials [1,2]. Electromagnetic and chemical enhancements, particularly electromagnetic field amplification (localized surface plasmon resonance (LSPR)) and charge transfer, contribute to Raman signal enhancement [3,4,5]. This dual-path enhancement elevates SERS sensitivity to single-molecule levels while retaining Raman spectroscopy advantages [6]. The pivotal aspect of SERS lies in enhancing substrates, notably plasmonic substrates, with Ag and Au being the most efficient due to their inherent plasmonic properties [7,8,9,10]. The substrate’s geometry, particularly the surface localization of nanostructures, is crucial for sustaining strong surface plasmon resonance (SPR), and the surface chemistry of these nanostructures significantly influences the intensity of SPR peaks [11,12,13,14,15]. At present, combining semiconductor nanostructures with noble metal nanoparticles, known as metal–semiconductor heterostructures, offers a promising approach to creating three-dimensional (3D) LSPR structures to enhance SERS substrates’ detection sensitivity and uniformity [16,17,18,19,20,21]. These heterostructures can serve as recyclable SERS substrates through the photocatalytic degradation of target molecules under UV light irradiation and facilitate charge transfer pathways for Raman scattering enhancement [22,23,24]. Among semiconductor nanostructures, titanium dioxide (TiO_2_) and zinc oxide (ZnO) nanostructures are popular due to their excellent photocatalytic activity when exposed to UV light, making them ideal SERS substrates [25,26,27]. Nevertheless, fewer studies explore the use of sodium titanate nanostructures in SERS and photocatalysis [28].

Sodium titanate (Na_2_Ti_n_O_2n+1_, where 2 ≤ n ≤ 9) has garnered considerable attention as a promising material due to its notable attributes: a high level of chemical inertness, non-toxicity, ion exchange capabilities, and cost-effectiveness [29,30,31]. This versatile substance finds applications in chemical absorption, photocatalysis, supercapacitors, and sodium-ion batteries [32,33,34,35,36,37]. Numerous synthesis methods have been employed to fabricate sodium titanate nanostructures [38,39,40]. The hydrothermal method has emerged as a preferred choice due to its simplicity and cost-effectiveness, making it widely used for obtaining a diverse range of nanostructures [35,41]. One-dimensional sodium titanate nanostructures adorned with Ag nanoparticles via an ion-sputtering technique have recently emerged as highly effective SERS substrates, showcasing exceptional detection sensitivity, stability, and uniformity [28]. This limitation stems from the necessity to conduct the deposition of Ag nanoparticles under high-vacuum conditions, which are energy-intensive and not environmentally friendly.

Prior investigations have often resorted to thermal evaporation or ion-sputtering techniques to achieve the uniform deposition of Au or Ag nanoparticles on one-dimensional semiconductor nanostructures to fabricate 3D SERS substrates [42,43,44]. These methods can effectively produce high-performance SERS substrates but fall short regarding energy savings and carbon reduction [45]. Moreover, the literature dedicated to the development of SERS substrates on one-dimensional semiconductor nanostructures through the application of a resorcinol–formaldehyde (RF) resin layer, a strategy aimed at diminishing the reliance on noble metal nanoparticles, has been relatively scant [46,47]. In earlier studies, resorcinol exhibited versatility by serving as a critical reactant in forming the RF layer and playing an essential role in passivating the surfaces of metal nanoparticles to prevent agglomeration [48,49]. Furthermore, resorcinol’s capabilities extended to acting as a reducing agent, facilitating the conversion of metal salts into their nanoparticle counterparts [50,51]. This innovative approach promises effective SERS substrates and aligns with sustainability goals by reducing the carbon footprint associated with traditional high-vacuum methods.

This study details the fabrication of Na_2_Ti_3_O_7_@RF@Ag heterostructures achieved through straightforward hydrothermal and wet chemical techniques, aiming to enhance their photocatalytic and SERS capabilities. The optimization of AgNO_3_ and L-ascorbic acid volumes was explored to maximize SERS enhancement, specifically for detecting rhodamine B (RhB) molecules. The Na_2_Ti_3_O_7_@RF@Ag heterostructures were thoroughly characterized to reveal their morphology, crystal structure, and chemical composition using microscopic and spectroscopic techniques. These heterostructures offer improved separation of photoelectron–hole pairs and increased surface-active sites, enhancing their photocatalytic efficiency and reusability. Additionally, the Na_2_Ti_3_O_7_@RF@Ag heterostructures present an advantageous geometry for the uniform deposition of Ag nanoparticles, facilitating the creation of high-performance SERS and photocatalytic substrates.

## 2. Results and Discussion

**Figure 1:**
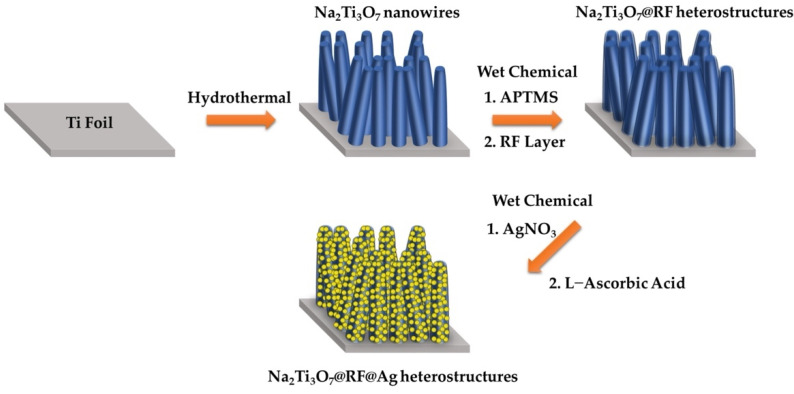
Synthetic scheme of the preparation of the Na_2_Ti_3_O_7_@RF@Ag heterostructures.

Figure 1 presents a schematic representation of the fabrication process for cultivating Na_2_Ti_3_O_7_@RF@Ag heterostructures. Initially, a straightforward hydrothermal technique was utilized to nurture Na_2_Ti_3_O_7_ nanowires on a Ti foil at 220 °C for 5 h. Subsequently, a self-assembled APTMS ((3-Aminopropyl)trimethoxysilane) monolayer was employed to enhance the hydrophilic properties of Na_2_Ti_3_O_7_ nanowires, allowing for the uniform decoration of the RF layer [47]. Lastly, Na_2_Ti_3_O_7_ nanowires coated with an RF layer, in conjunction with L-ascorbic acid, facilitated the reduction of Ag^+^ to Ag, culminating in the formation of Na_2_Ti_3_O_7_@RF@Ag heterostructures.

**Figure 2:**
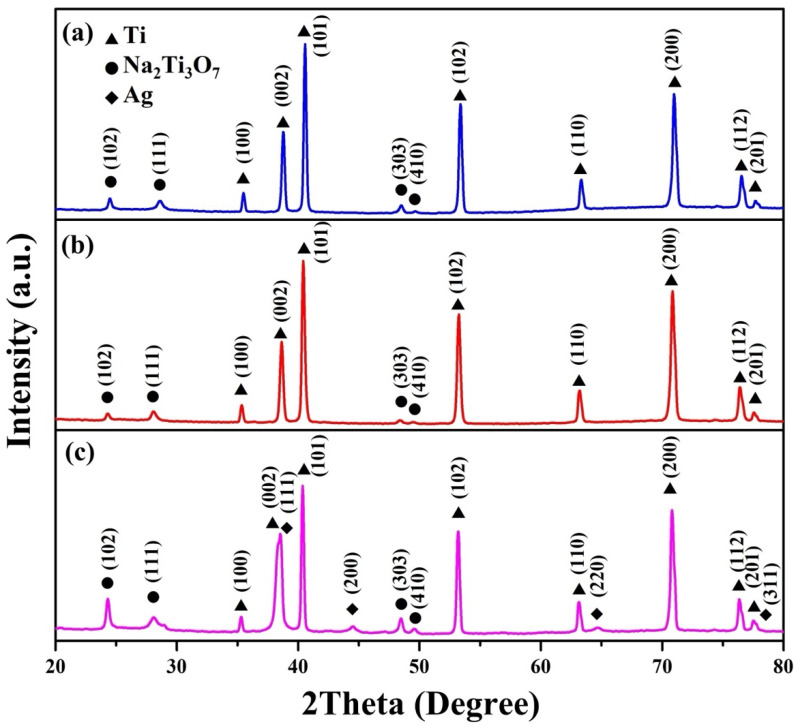
XRD spectra of (**a**) Na_2_Ti_3_O_7_ nanowires, (**b**) Na_2_Ti_3_O_7_@RF heterostructures, and (**c**) Na_2_Ti_3_O_7_@RF@Ag heterostructures.

Figure 2 illustrates the X-ray powder diffraction (XRD) patterns of the Na_2_Ti_3_O_7_ nanowires, Na_2_Ti_3_O_7_@RF heterostructures, and Na_2_Ti_3_O_7_@RF@Ag heterostructures. The XRD patterns for the Na_2_Ti_3_O_7_ nanowires (Figure 2a) exhibit distinct peaks at 2θ values of 24.3°, 28.5°, 48.5°, and 49.5°, corresponding to the (102), (111), (303), and (410) crystal planes of monoclinic-phase Na_2_Ti_3_O_7_ (JCPDS Card No. 72-0148), respectively. Additionally, these peaks are observed at 2θ angles of 35.1°, 38.4°, 40.2°, 53.0°, 62.9°, 70.7°, 74.2°, 76.2°, and 77.4°, corresponding to the (100), (002), (101), (102), (110), (103), (200), (112), and (201) crystal planes of hexagonal-phase Ti (JCPDS Card No. 44-1294), respectively. Figure 2b shows the XRD pattern of Na_2_Ti_3_O_7_@RF heterostructures of Na_2_Ti_3_O_7_ nanowires without any distinctive peaks. Notably, the presence of Ag nanoparticles within the Na_2_Ti_3_O_7_@RF@Ag heterostructures (Figure 2c) is evident from the faint peaks at 2θ values of 38.2°, 44.4°, 64.6°, and 77.6°, corresponding to the (111), (200), (220), and (311) crystal planes of cubic-phase Ag (JCPDS Card No. 87-0720). This outcome verifies the absence of impurities within the Na_2_Ti_3_O_7_@RF@Ag heterostructures.

**Figure 3:**
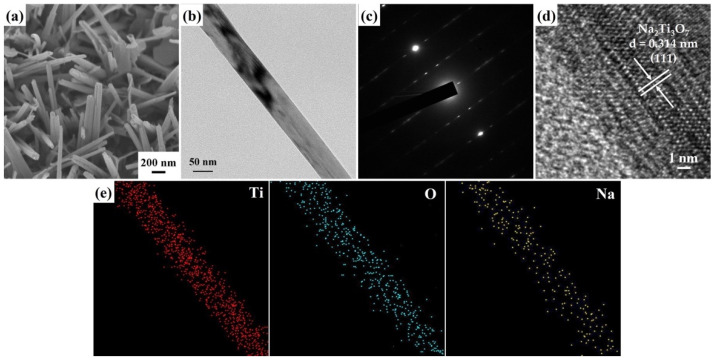
(**a**) A 45° tilt-view FESEM image of the Na_2_Ti_3_O_7_ nanowires. (**b**) FETEM image, (**c**) SAED pattern, (**d**) HRTEM image, and (**e**) FETEM-EDS mapping image of a Na_2_Ti_3_O_7_ nanowire.

The FESEM morphology (Figure 3a) of the Na_2_Ti_3_O_7_ nanowires reveals a high-density distribution of these nanowires that have grown across the entire surface of the Ti foil, displaying a random orientation. Figure 3b displays a FETEM image of a Na_2_Ti_3_O_7_ nanowire featuring an intermediate diameter of approximately 105 nm. Additionally, Figure 3c reveals a selected area electron diffraction (SAED) pattern of a Na_2_Ti_3_O_7_ nanowire, presenting a well-defined arrangement of single crystal dots. This single-crystal-dot array corresponds to the crystal structure of the Na_2_Ti_3_O_7_ nanowire and aligns with the monoclinic Na_2_Ti_3_O_7_ crystal phase (JCPDS Card No. 72-0148). The HRTEM image (Figure 3d) demonstrates an index lattice spacing of 0.314 nm, which agrees with the lattice spacing of the (111) plane of Na_2_Ti_3_O_7_. In addition, the FETEM-EDS mapping images (Figure 3e) prove the homogeneous distribution of Ti, O, and Na elements in the nanowire. The calculated atomic percentages of the Na_2_Ti_3_O_7_ nanowires from the FETEM-EDS mapping images reveal the following compositions: Na 7.9%, Ti 25.1%, and O 67%.

**Figure 4:**
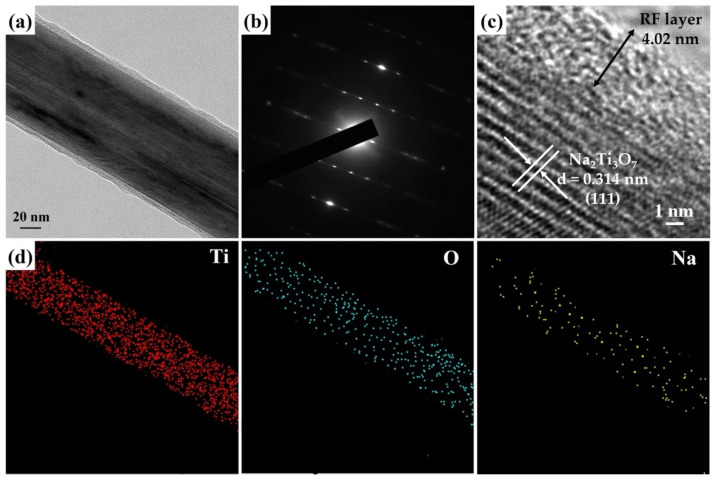
(**a**) FETEM image, (**b**) SAED pattern, (**c**) HRTEM image, and (**d**) FETEM-EDS mapping image of a Na_2_Ti_3_O_7_@RF heterostructure.

Figure 4a reveals an FETEM image of a Na_2_Ti_3_O_7_@RF heterostructure with a core–shell structure. Figure 4b shows an SAED pattern of a Na_2_Ti_3_O_7_@RF heterostructure which only presents a single-crystal-dot array aligned with the monoclinic Na_2_Ti_3_O_7_ crystal phase (JCPDS Card No. 72-0148). The HRTEM image (Figure 4c) reveals the presence of an approximately 4.02 nm thick amorphous RF layer decorated on the Na_2_Ti_3_O_7_ nanowire. Additionally, a lattice fringe characterized by an interplanar spacing of 0.314 nm corresponds to the (111) plane of the monoclinic Na_2_Ti_3_O_7_ crystal phase (JCPDS Card No. 72-0148). The FETEM-EDS mapping images (Figure 4d) still exhibit the heterostructure’s homogeneous Ti, O, and Na distribution. This observation verifies that the presence of the RF layer on the Na_2_Ti_3_O_7_ nanowire does not induce any alterations in the material’s elemental composition.

**Figure 5:**
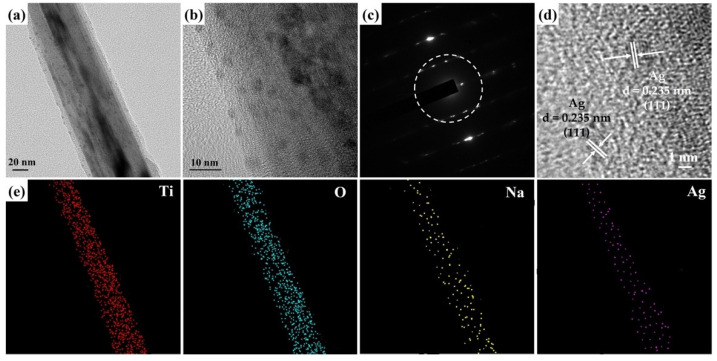
(**a**,**b**) FETEM image, (**c**) SAED pattern, (**d**) HRTEM image, and (**e**) FETEM-EDS mapping image of a Na_2_Ti_3_O_7_@RF@Ag heterostructure (0.15 mL of Ag NO_3_ and 3 mL of L-ascorbic acid).

Figure 5a,b presents the FETEM images of a Na_2_Ti_3_O_7_@RF@Ag heterostructure grown using 0.15 mL AgNO_3_ and 3 mL L-ascorbic acid volumes. The FETEM images can reveal that the Ag nanoparticles’ size is about 3–10 nm, and they are completely decorated on the Na_2_Ti_3_O_7_@RF heterostructure. The SAED pattern (Figure 5c) of a Na_2_Ti_3_O_7_@RF@Ag heterostructure displays a combination of a single-crystal-dot array and a polycrystalline ring. The presence of a single-crystal-dot array in the SAED pattern of Na_2_Ti_3_O_7_@RF@Ag heterostructure corresponds to the crystalline structure of Na_2_Ti_3_O_7_ nanowire, which aligns with the monoclinic Na_2_Ti_3_O_7_ crystal phase (JCPDS Card No. 72-0148). This alignment indicates the well-preserved crystal structure of Na_2_Ti_3_O_7_ even after the deposition of Ag nanoparticles. The Ag nanoparticles’ distinctive appearance exhibited a concentric ring pattern, corresponding to the diffraction from the (111) plane of the cubic Ag crystal structure (JCPDS Card No. 87-0720). The HRTEM image of the Na_2_Ti_3_O_7_@RF@Ag heterostructure (Figure 5d) shows two visible lattice fringes exhibiting an interplanar spacing of 0.235 nm which can be confidently attributed to the (111) lattice plane of the cubic Ag (JCPDS Card No. 87-0720). This observation underscores the successful deposition of cubic-phase Ag nanoparticles within the heterostructures. The FETEM-EDS mapping image (Figure 5d) provides valuable insights into the composition of the Na_2_Ti_3_O_7_@RF@Ag heterostructure. This composition analysis reveals the presence of Ti, O, Na, and Ag elements within the Na_2_Ti_3_O_7_@RF@Ag heterostructure, indicating the successful synthesis of this heterostructure through a two-step wet chemical process on the Na_2_Ti_3_O_7_ nanowire.

**Figure 6:**
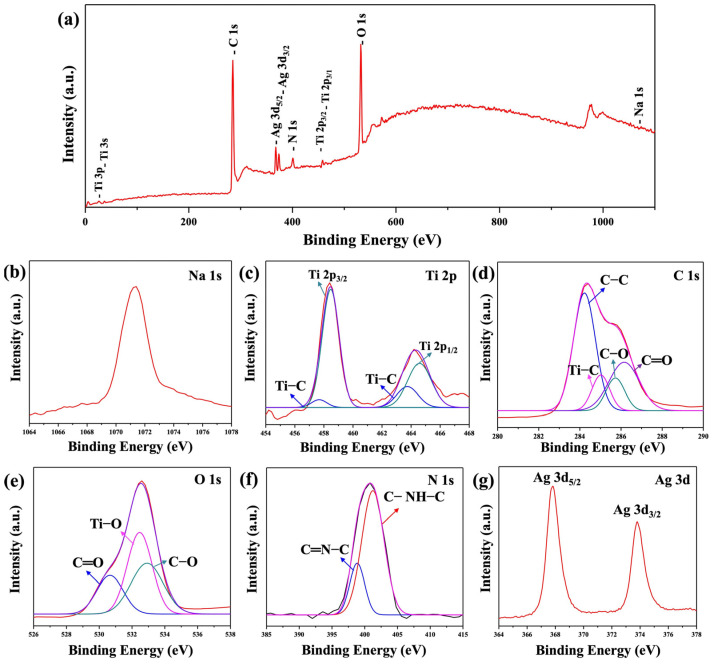
XPS spectra of the Na_2_Ti_3_O_7_@RF@Ag heterostructures (0.15 mL Ag NO_3_ and 3 mL L-ascorbic acid): (**a**) survey spectrum, (**b**) Na 1s, (**c**) Ti 2p, (**d**) C 1s, (**e**) O 1s, (**f**) N 1s, and (**g**) Ag 3d, respectively.

X-ray photoelectron spectroscopy (XPS) is a powerful tool for investigating the chemical states of elements in Na_2_Ti_3_O_7_@RF@Ag heterostructures (0.15 mL AgNO_3_ and 3 mL L-ascorbic acid volumes), providing valuable insights into surface chemistry. The XPS survey spectrum (Figure 6a) shows the existence of Na, Ti, C, O, N, and Ag in the Na_2_Ti_3_O_7_@RF@Ag heterostructures. The high-resolution (HR) XPS spectrum of Na 1s (Figure 6b) exhibits a peak at 1071.4 eV, confirming the valence state of Na as +1 [52,53]. The presence of Ti^4+^ species is confirmed by Ti 2p doublets with binding energies of 458.5 eV and 464.6 eV, corresponding to Ti 2p_3/2_ and Ti 2p_1/2_, respectively, as shown in Figure 6c. Two additional minor peaks were detected at 459.1 and 463.0 eV, consistent with the presence of the Ti–C bond, confirming the robust adhesion of the RF layer to the surface of the Na_2_Ti_3_O_7_ [54]. The C 1s spectrum of the RF layer (Figure 6d) is deconvoluted, revealing four distinct peaks centered at 284.2, 285.0, 285.7, and 286.1 eV which can be attributed to C–C, Ti–C, C–O, and C=O bonds, respectively [55,56]. The O 1s spectrum of the RF layer (Figure 6e) can be successfully deconvoluted with three discernible peaks at 530.6, 532.5, and 532.9 eV which are assigned to the C=O, Ti–O, and C–O groups, respectively [56,57]. The N 1s spectrum of the RF layer (Figure 6f) can also successfully deconvolute into two distinct peaks at 398.4 eV and 401.2.6 eV, corresponding to C-NH-C and C=N-C, respectively [58]. The HRXPS spectrum of Ag 3d (Figure 6g) can be observed at binding energies of 367.8 and 373.8 eV, corresponding to Ag 3d_5/2_ and Ag 3d_3/2_, respectively. The 6 kV binding energy difference between these peaks confirms the presence of an Ag^0^ state [59].

**Figure 7:**
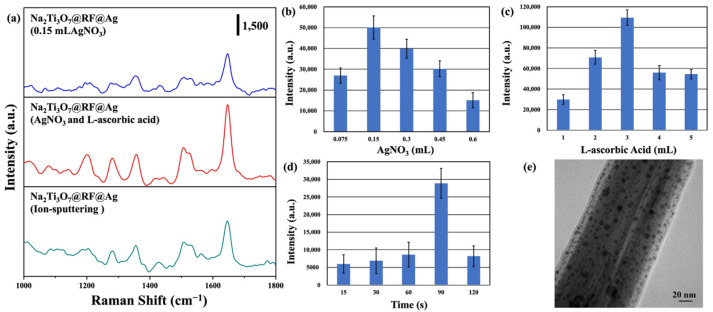
(**a**) SERS spectra of (10^−6^ M) RhB solution on Na_2_Ti_3_O_7_@RF@Ag heterostructures (only 0.15 mL of AgNO_3_), Na_2_Ti_3_O_7_@RF@Ag heterostructures (0.15 mL of AgNO_3_ and 3 mL of L-ascorbic acid), and Na_2_Ti_3_O_7_@RF@Ag heterostructure (Ag-ion-sputtering time of 90 s). The SERS intensities of (10^−6^ M) RhB solution on Na_2_Ti_3_O_7_@RF@Ag heterostructures at 1647 cm^−1^ under the different (**b**) AgNO_3_ volumes, (**c**) L-ascorbic acid volumes, and (**d**) Ag-ion-sputtering times. (**e**) FETEM image of a Na_2_Ti_3_O_7_@RF@Ag heterostructure (Ag-ion-sputtering time of 90 s).

SERS activity is intricately linked to the morphology, size, and density of Au and Ag nanoparticles, which collectively govern the efficiency and performance of SERS measurements [60,61]. This investigation assessed the SERS performance of the Na_2_Ti_3_O_7_@RF@Ag heterostructures synthesized under various reaction conditions, using RhB as the target molecule. Figure 7a shows the SERS spectra of Na_2_Ti_3_O_7_@RF@Ag heterostructures grown via different Ag-nanoparticle-deposition methods, such as the wet chemical method (only 0.15 mL of AgNO_3_; 0.15 mL of AgNO_3_ and 3 mL of L-ascorbic acid) and ion-sputtering method (Ag-ion-sputtering time of 90 s), which were immersed in the RhB solution (10^−6^ M) for 1h. The primary vibrational modes for the characteristic peaks of the RhB molecule are aromatic C–C stretching (1076 cm^−1^), C-H in-plane bending (1197 cm^−1^), C–H bending (1279 cm^−1^), the stretching vibration of bridge C–C aromatic bonds (1360 cm^−1^), aromatic C–C bending (1508 cm^−1^), C–H stretching (1527 cm^−1^), aromatic C-C bending, and C=C stretching vibration (1647 cm^−1^), respectively [62,63]. This study chose a Raman peak at 1647 cm^−1^ as the reference point to assess SERS activity because it does not readily overlap with the SERS substrate or the solvent. The Raman intensity of RhB is significantly boosted in the presence of Na_2_Ti_3_O_7_@RF@Ag heterostructures (0.15 mL of AgNO_3_ and 3 mL of L-ascorbic acid) compared to the Na_2_Ti_3_O_7_@RF@Ag heterostructures (only 0.15 mL of AgNO_3_) and the Na_2_Ti_3_O_7_@RF@Ag heterostructure (Ag-ion-sputtering time of 90 s). The efficacy of the Na_2_Ti_3_O_7_@RF@Ag heterostructures for RhB detection was assessed using the SERS enhancement factor (EF), determined by the formula EF = I_SERS_ × C_0_/I_0_ × C_SERS_. Here, I_SERS_ and I_0_ represent the SERS (with Na_2_Ti_3_O_7_@RF@Ag heterostructures) and standard Raman intensities of RhB, while C_SERS_ and C_0_ denote the RhB concentration on the Na_2_Ti_3_O_7_@RF@Ag heterostructures (10^−6^ M) and bare Ti foil (10^−4^ M) [64,65]. The calculated maximum EF values for the RhB Raman band at 1647 cm^−1^ are 5.00 × 10^6^ (0.15 mL AgNO_3_), 1.09 × 10^7^ (0.15 mL of AgNO_3_ and 3 mL of L-ascorbic acid), and 2.88 × 10^6^ (Ag-ion-sputtering time of 90 s), respectively. The Na_2_Ti_3_O_7_@RF@Ag heterostructures (0.15 mL AgNO_3_ and 3 mL L-ascorbic acid) are 2.18 and 3.78 times higher than the Na_2_Ti_3_O_7_@RF@Ag heterostructures (0.15 mL of AgNO_3_) and Na_2_Ti_3_O_7_@RF@Ag heterostructures (Ag-ion-sputtering time of 90 s), respectively.

Figure 7b shows the SERS intensity of an RhB solution (10^−6^ M) at 1647 cm^−1^ obtained from Na_2_Ti_3_O_7_@RF@Ag heterostructures grown using different volumes of AgNO_3_. The most prominent Raman intensity is observed at 0.15 mL of AgNO_3_. As the volume of AgNO_3_ increases, there is a gradual decrease in the Raman intensity. As the SERS enhancement effect remained relatively low, this research introduced different volumes of L-ascorbic acid to expedite the formation and deposition of Ag nanoparticles, as shown in Figure 7c. The most prominent Raman signal is observed for 3 mL of L-ascorbic acid. This trend may be attributed to the more effective distribution of hot spots achieved with 3 mL of L-ascorbic acid, significantly enhancing Raman signal intensity. This result is consistent with previous FETEM observations. To confirm the effectiveness of the Ag nanoparticle preparation method employed in the wet chemical method compared to the ion-sputtering method, this study used the ion-sputtering method to deposit Ag nanoparticles onto Na_2_Ti_3_O_7_@RF heterostructures at different times. Figure 7d reveals the SERS intensity of an RhB solution (10^−6^ M) at 1647 cm^−1^ obtained from Na_2_Ti_3_O_7_@RF@Ag heterostructures grown using different Ag-ion-sputtering times. Hence, it can be determined that the most significant SERS enhancement effect was achieved with an ion-sputtering time of 90 s. This result is much lower than for Na_2_Ti_3_O_7_@RF@Ag heterostructures prepared via wet chemical methods (0.15 mL of AgNO_3_ and 3 mL of L-ascorbic acid). In order to understand the differences, Na_2_Ti_3_O_7_@RF@Ag heterostructures (Ag-ion-sputtering time of 90 s) were further analyzed through FETEM. Figure 7e displays a FETEM image that depicts a size range of Ag nanoparticles, varying from approximately 3 to 21 nm. While the Ag nanoparticles are thoroughly dispersed within the Na_2_Ti_3_O_7_@RF heterostructure, their size distribution appears irregular. This non-uniformity in size has consequences for the hot spot distribution on the heterostructure’s surface, reducing its SERS enhancement effect [24,66]. Hence, the simplicity and remarkable efficiency of this study’s Ag nanoparticle preparation method can be further validated.

**Figure 8:**
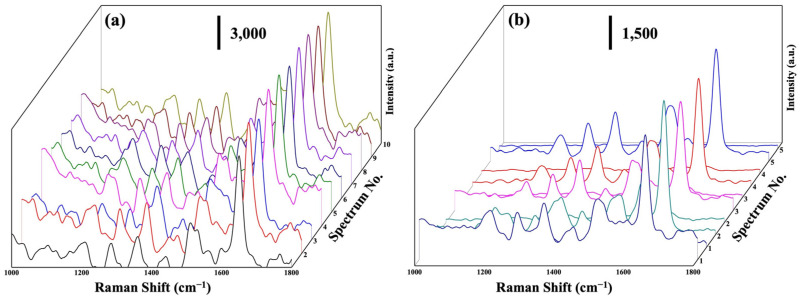
(**a**) SERS spectra of a (10^−6^ M) RhB solution at 10 random points on Na_2_Ti_3_O_7_@RF@Ag heterostructures. (**b**) SERS spectra of a (10^−7^ M) RhB solution on Na_2_Ti_3_O_7_@RF@Ag heterostructures for the five cycles.

The uniformity and reusability of SERS substrates are crucial factors in real-world SERS applications [25,47]. In order to assess the uniformity of the Na_2_Ti_3_O_7_@RF@Ag heterostructures, ten random spots were chosen on the substrate loaded with RhB solution (10^−6^ M) for an SERS analysis. Figure 8a shows the SERS spectra of an RhB solution (10^−6^ M) acquired from these ten positions on the substrate. The Raman signals’ peak positions and intensities exhibit remarkable uniformity across various locations on the the Na_2_Ti_3_O_7_@RF@Ag heterostructures. This outcome further substantiates the effective enhancement of Raman signals achieved by the Na_2_Ti_3_O_7_@RF@Ag heterostructures. The reusability of the Na_2_Ti_3_O_7_@RF@Ag heterostructures is a pivotal concern in this study. In order to address this issue, RhB molecules were systematically eliminated from the Na_2_Ti_3_O_7_@RF@Ag heterostructures using photocatalytic degradation facilitated by exposure to UV light for 1h. Figure 8b reveals the Raman spectra of Na_2_Ti_3_O_7_@RF@Ag heterostructures initially immersed in an RhB solution with a 10^−7^ M concentration for 1 h. These spectra are presented before and after five consecutive UV light exposure cycles. These observations emphasize the remarkable reusability of the Na_2_Ti_3_O_7_@RF@Ag heterostructures, which consistently retain a comparable Raman signal strength even after undergoing five repeated cycles. This result reconfirms their enduring efficacy and suitability for practical applications. In addition, this occurrence can be attributed to the unique structural configuration of the Na_2_Ti_3_O_7_@RF@Ag heterostructures, which provide an augmented quantity of hot spots and surface-active sites. As a result, Na_2_Ti_3_O_7_@RF@Ag heterostructures exhibit remarkable SERS enhancement, excellent reproducibility, and sustained stability over an extended period.

**Figure 9:**
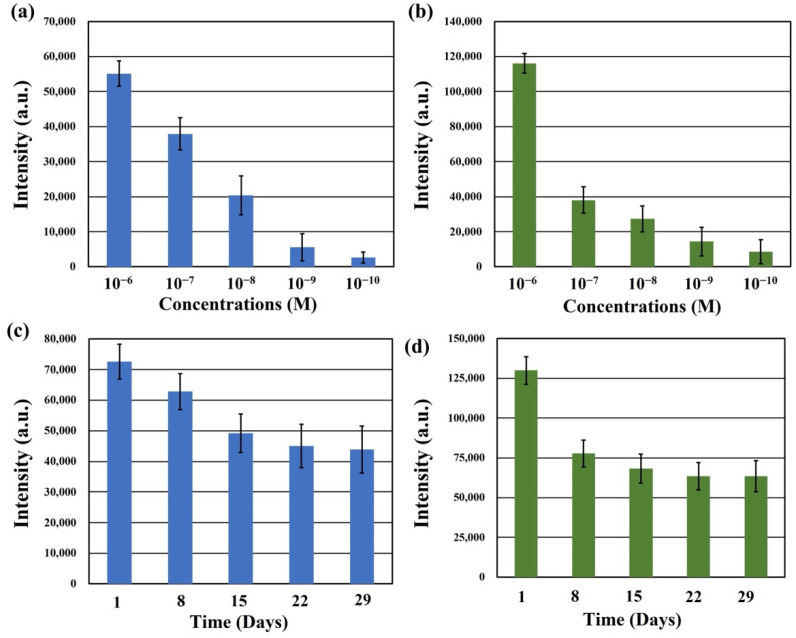
The SERS intensities of Na_2_Ti_3_O_7_@RF@Ag heterostructures at (**a**) 1647 cm^−1^ (RhB) and (**b**) 1623 cm^−1^ (MB) under different concentrations of RhB and MB solutions, respectively. (**b**) The SERS intensities of Na_2_Ti_3_O_7_@RF@Ag heterostructures at (**c**) 1647 cm^−1^ (RhB) and (**d**) 1623 cm^−1^ (MB) under different detection times.

The SERS spectra were acquired for freshly synthesized SERS substrates after their immersion in different concentrations of RhB solution for 1 h, followed by air-drying. This assessment aimed to determine the low detection limit of the Na_2_Ti_3_O_7_@RF@Ag heterostructures for RhB solutions. Figure 9a displays the SERS intensities of the Na_2_Ti_3_O_7_@RF@Ag heterostructures at 1647 cm^−1^ over a concentration range from 10^−6^ M to 10^−10^ M of RhB solution. As the RhB concentration decreases, the SERS signal intensities at 1647 cm^−1^ gradually diminish. When the RhB solution concentration decreases below 10^−10^ M, the SERS signal at 1647 cm^−1^ becomes challenging to effectively detect for Na_2_Ti_3_O_7_@RF@Ag heterostructures. Nevertheless, even at a concentration as low as 10^−10^ M, the SERS signal at 1647 cm^−1^ can still be reliably detected using Na_2_Ti_3_O_7_@RF@Ag heterostructures. To ascertain the versatility of the Na_2_Ti_3_O_7_@RF@Ag heterostructure across various organic dyes, this study investigated the SERS effect of different methylene blue (MB) concentrations. Figure 9b displays the SERS intensities of Na_2_Ti_3_O_7_@RF@Ag heterostructures at 1623 cm^−1^ over a concentration range from 10^−6^ M to 10^−10^ M of MB solution. As the concentration of the MB solution drops below 10^−10^ M, effectively detecting the SERS signal at 1623 cm^−1^ becomes challenging for Na_2_Ti_3_O_7_@RF@Ag heterostructures.

In order to evaluate the temporal stability of the Na_2_Ti_3_O_7_@RF@Ag heterostructures, RhB and MB solutions, each with a concentration of 10^−6^ M, were analyzed after preparing the SERS substrate and storing it in the dark for different durations of 1, 8, 15, 22, and 29 days. Figure 9c,d present the SERS intensities of Na_2_Ti_3_O_7_@RF@Ag heterostructures at 1647 cm^−1^ (RhB) and 1623 cm^−1^ (MB) for these different detection times, respectively. Remarkably, even after 29 days of storage, the intensities of the SERS signal at 1647 cm^−1^ (RhB) and 1623 cm^−1^ (MB) retain substantial levels of 60.4% and 48.9%, respectively. This result can verify that the Na_2_Ti_3_O_7_@RF@Ag heterostructures exhibit long-term stability.

**Figure 10:**
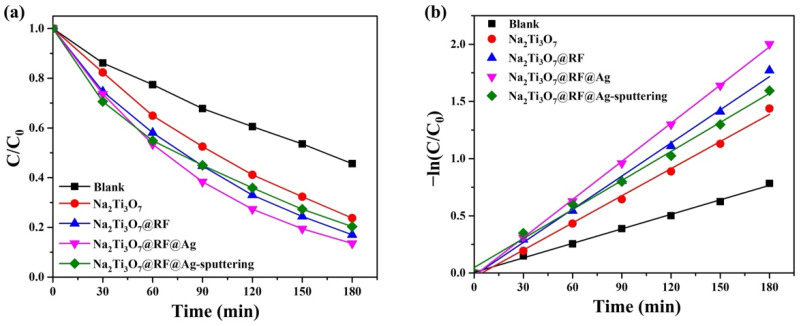
(**a**) Photocatalytic activities and (**b**) kinetic linear simulation curves of as-prepared photocatalysts under UV light irradiation.

The primary absorption peak at 552 nm for RhB gradually diminishes as the duration of UV light irradiation increases [67,68]. Notably, there is no shift in the primary peak of RhB, indicating that the process primarily involves decomposing the benzene/heterocyclic rings [69]. In order to assess photocatalytic activity, a plot of (C/C_0_) versus time is presented in Figure 10a. The photocatalytic efficiency values of various samples, including Na_2_Ti_3_O_7_ nanowires, Na_2_Ti_3_O_7_@RF heterostructures, Na_2_Ti_3_O_7_@RF@Ag heterostructures (0.15 mL of AgNO_3_ and 3 mL of L-ascorbic acid), and Na_2_Ti_3_O_7_@RF@Ag heterostructures (Ag-ion-sputtering of 90 s), are 76.3, 83.0, 86.5, and 79.7%, respectively. The Langmuir–Hinshelwood model is widely employed to assess the photodegradation kinetics of organic dyes in aqueous solutions. The reaction-rate constants (k) for different samples, including Na_2_Ti_3_O_7_ nanowires, Na_2_Ti_3_O_7_@RF heterostructures, Na_2_Ti_3_O_7_@RF@Ag heterostructures (0.15 mL AgNO_3_ and 3 mL L-ascorbic acid), and Na_2_Ti_3_O_7_@RF@Ag heterostructures (Ag-ion-sputtering of 90 s), are determined to be 0.0079, 0.00966, 0.01112, and 0.00846 min^−1^, respectively, as depicted in Figure 10b. These findings suggest that the Na_2_Ti_3_O_7_@RF@Ag heterostructures (0.15 mL of AgNO_3_ and 3 mL of L-ascorbic acid) exhibited approximately 1.4 and 1.3 times greater efficiency compared to Na_2_Ti_3_O_7_ nanowires and Na_2_Ti_3_O_7_@RF@Ag heterostructures (Ag-ion-sputtering of 90 s). The Na_2_Ti_3_O_7_@RF@Ag heterostructures reveal exceptional efficiency as reusable photocatalysts for the photodecomposition of RhB solutions under UV light irradiation. This outcome can be ascribed to the heightened suppression of electron–hole pair recombination and the increased availability of surface-active sites.

**Figure 11:**
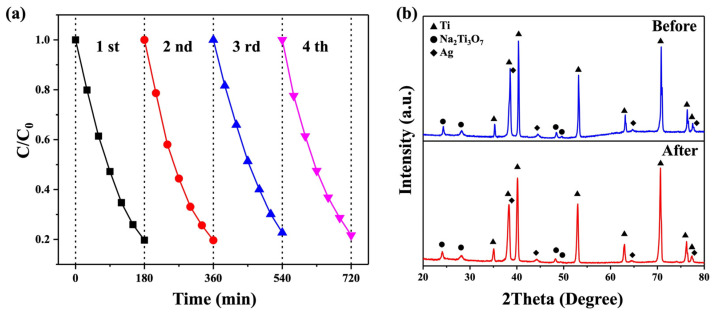
(**a**) The reusability of the Na_2_Ti_3_O_7_@RF@Ag heterostructures under UV light irradiation. (**b**) XRD spectra of Na_2_Ti_3_O_7_@RF@Ag heterostructures before cycling and after the fourth cycle.

The reusability of photocatalysts is of the utmost importance as it directly impacts their effectiveness in photocatalytic processes. In this study, an RhB solution with Na_2_Ti_3_O_7_@RF@Ag heterostructures (0.15 mL of AgNO_3_ and 3 mL of L-ascorbic acid) was subjected to UV light irradiation for 3 h. Subsequently, the substrate was thoroughly rinsed twice with copious amounts of de-ionized water in preparation for the next cycle of photodegradation. Over four cycles, the photocatalytic efficiency values of the Na_2_Ti_3_O_7_@RF@Ag heterostructures on Ti foil were found to be 80.3, 80.4, 77.4, and 78.3, respectively, as depicted in Figure 11a. The results reveal that the Na_2_Ti_3_O_7_@RF@Ag heterostructures exhibit superior reusability for decomposing the RhB solution. Furthermore, an XRD analysis of Na_2_Ti_3_O_7_@RF@Ag heterostructures (0.15 mL of AgNO_3_ and 3 mL of L-ascorbic acid) after undergoing four cycles of recycling (Figure 11b) indicate no significant crystalline alterations, substantiating the enduring nature of the heterojunction. This analysis underscores the exceptional and sustained photocatalytic degradation efficiency of Na_2_Ti_3_O_7_@RF@Ag heterostructures.

## 3. Materials and Methods

### 3.1. Materials

A 0.25 mm thick titanium (Ti) foil (99.5%) was commercially sourced from Alfa Aesar (Haverhill, MA, USA). All chemicals used were obtained from commercial suppliers and were employed without the need for further purification. Specifically, hydrochloric acid (HCl, 37%), sodium hydroxide (NaOH, 97%), ethanol (C_2_H_5_OH, 99%), L-ascorbic acid (C_6_H_8_O_6_, 99%), and rhodamine B (RhB, C_28_H_31_ClN_2_O_3_, 95%) were acquired from Sigma-Aldrich (Steinheim, Germany). (3-Aminopropyl)trimethoxysilane (C_6_H_17_NO_3_Si, APTMS, 95%) was acquired from Acros (Renningen, Germany). Resorcinol (C_6_H_6_O_2_, 99%), ammonium hydroxide (NH_4_OH, 28%), methylene blue (MB, C_16_H_18_ClN_3_S, 95%), and silver nitrate (AgNO_3_, 99%) were obtained from Alfa Aesar (USA). A formaldehyde solution (CH_2_O, 37%) was acquired from Merck (Darmstadt, Germany). De-ionized water with a resistivity exceeding 18.2 MΩ was used to prepare all solutions.

### 3.2. Syntheses of Na_2_Ti_3_O_7_ Nanowires

A 0.25 mm thick titanium (Ti) foil was cut to the desired dimensions 0.5 cm × 0.5 cm or 1.5 cm × 2.5 cm. The substrate underwent ultrasonic treatment in ethanol and 1.0 M HCl for 10 min each, effectively removing organic contaminants and oxide layers. After each treatment, it was thoroughly rinsed with ethanol and dried using an air purge. Subsequently, the cleaned substrate was placed in separate 50 mL Teflon-lined stainless-steel autoclaves containing a 0.375 M NaOH solution (20 mL) and heated to 220 °C for 5 h. Finally, the substrate was washed with de-ionized water and ethanol and dried via air purge.

### 3.3. Syntheses of Na_2_Ti_3_O_7_@RF@Ag Heterostructures

The substrates featuring Na_2_Ti_3_O_7_ nanowires underwent a surface hydrophilicity enhancement process by immersing them in a 50 mL ethanol solution containing 5 mM APTMS for 6 h at room temperature. Subsequently, the substrates were thoroughly rinsed with ethanol and de-ionized water and dried using an air purge. For the growth of the RF layer on the Na_2_Ti_3_O_7_ nanowires, the substrates were placed in a 40 mL aqueous solution containing 16.37 mg of resorcinol, 0.0284 mL of NH_4_OH, and 0.0444 mL of CH_2_O. This mixture was vigorously stirred for 6 h at 50 °C. Next, the substrate was rinsed with ethanol thrice and dried at 60 °C for 2 h. To deposit Ag nanoparticles on the Na_2_Ti_3_O_7_@RF heterostructures, the substrate was immersed in a 50 mL aqueous solution with different volumes of AgNO_3_ (5 mM) and L-ascorbic acid (50 mM) under vigorous stirring for 1 h. Finally, the resulting substrate underwent several ethanol rinses and was dried at 60 °C for 2 h.

### 3.4. Characterization

The as-prepared SERS substrates underwent a comprehensive analysis to investigate their microstructures and elemental composition. Field-emission scanning electron microscopy (FESEM) was employed using a Hitachi S-4800 instrument (Tokyo, Japan). Field-emission transmission electron microscopy (FETEM) was also utilized with a JEOL JSM-2100F apparatus (Tokyo, Japan) equipped with energy-dispersive X-ray spectroscopy (EDS). X-ray diffraction (XRD) was performed using a Bruker (Billerica, MA, USA) D8 SSS instrument based in the United States to characterize the crystal structures of the as-prepared substrates. The chemical states of the elements within the Na_2_Ti_3_O_7_@RF@Ag heterostructures were determined through X-ray photoelectron spectroscopy (XPS), conducted using a ULVAC-PHI PHI 5000 VersaProbe instrument (Chigasaki, Japan). Furthermore, SERS measurements were carried out using a micro-Raman identify spectrometer (MRI532S) provided by Protrustech in Taiwan. These measurements utilized an excitation wavelength of 532 nm, with a laser beam diameter of 3.3 mm and a resolution of 2.2 cm⁻^1^.

### 3.5. SERS and Photocatalytic Measurement

For SERS measurement, a micro-Raman identify spectrometer with 532 nm excitation wavelength employed 1 mW laser power and 0.15 s detector integration time. The SERS properties of the Na_2_Ti_3_O_7_@RF@Ag heterostructures were evaluated, immersing substrates in RhB solutions and drying them for 1 h at room temperature in the dark. For photocatalytic measurements, photocatalyst activity was assessed by degrading an RhB solution (0.08 mM) without pH adjustment under UV light (253.7 nm, 10 W, Philips, Amsterdam, The Netherlands) irradiation. Concentrations were measured with a DR/UV-Vis spectrometer (Hitachi U-2900, Tokyo, Japan), and efficiency was calculated as C/C_0_, where C_0_ and C represent initial and final RhB concentrations.

## 4. Conclusions

A 3D Na_2_Ti_3_O_7_@RF@Ag heterostructure is synthesized through a multi-step process featuring Na_2_Ti_3_O_7_ nanowire cores, an intermediate RF layer, and outer Ag nanoparticle sheaths. Initially, single-crystal Na_2_Ti_3_O_7_ nanowires are grown directly on flexible Ti foil using a one-step hydrothermal technique. Subsequently, a two-step wet chemical process deposits the RF layer and Ag nanoparticles on the nanowires at a low temperature. By optimizing AgNO_3_ and L-ascorbic acid concentrations, the resulting Na_2_Ti_3_O_7_@RF@Ag heterostructures exhibit higher surface-enhanced Raman scattering (SERS) enhancement for detecting RhB molecules. The unique geometry of these heterostructures, providing numerous hot spots and surface-active sites, contributes to significant SERS enhancement, remarkable reproducibility, and long-term stability. These Na_2_Ti_3_O_7_@RF@Ag heterostructures efficiently serve as reusable photocatalysts for decomposing RhB solutions under UV light irradiation, benefitting from the enhanced inhibition of electron–hole pair recombination and an increased number of surface-active sites. This dual functionality highlights the versatility of Na_2_Ti_3_O_7_@RF@Ag heterostructures, promising applications in environmental remediation and chemical sensing.

## Data Availability

Data are contained within the article.
